# Traumatic Tetanus

**Published:** 1854-06

**Authors:** W. K. Bowling

**Affiliations:** Nashville, Tenn.


### *Traumatic Tetanus.*

BY W. K. BOWLING, M. D., OF NASHVILLE, TENN.

Dr. Carpenter, of Patchogue, Suffolk county, Long Island, reports two cases of Traumatic Tetanus in the Ohio Med. and Surg. Journal, "successfully treated by ice." On the 25th of January last, we were called some eight miles into the country by Dr. H., a wealthy and retired physician, to see a negro man belonging to the doctor, afflicted with Traumatic Tetanus. The patient was a stout, muscular man, about 30 years of age, and at the time of the injury, was in excellent health. He was engaged in hauling poles upon a wagon, one of which, in unloading, fell upon the great toe of the right foot, lacerating it considerably, and producing great pain. He was sent to his cabin, and some domestic remedies applied. For two weeks he kept his room, and reported his toe every day to be improving.—Finally, news reached his master that the man "had something like a fit." The doctor visited him, discovered considerable dyspnœa, quickened and hard pulse, and learned that the bowels were confined, and that the patient complained of some pain in the chest. He thought it possible the boy had pneumonia, and immediately bled him and gave him a mercurial cathartic. On the next day, there was a noticeable rigidity of the muscles of the neck and face, with difficulty of deglutition. The mouth could be opened, but with great difficulty, and the abdominal muscles soon became as rigid and hard as a board. The rigidity, while tonic—permanent—was increased temporarily by the slightest touch of the patient, painfully jerking the body, interrupting the respiratory movement, and occasioning the short grunt common in pleuritic cases. At this period of his disease we saw him with the doctor. The patient's face was peculiarly tetanic in its expression, which, together with the history, and the absence of all physical signs of pneumonia or pleurisy, made the diagnosis but too apparent. The pulse was 120 to the minute, and pretty firm ; and the mercurial, administered by the doctor, though followed by castor and turpentine oil, had failed to move his bowels. Large injections, however, of soap and water, soon procured free evacuations ; and with them, as we had often observed in similar cases, a rapid increase of spasm, affecting a large portion of the muscular system not previously involved. The muscles of the back became rigidly affected, drawing the body forcibly backwards. The agony started large drops of sweat, which beaded his forehead, face and chest. He was taken out of the bed, now defiled with the discharges, and moved to another in the same room. We had already dispatched a messenger to the ice-house for ice, which, being at hand, as soon as he was again in bed, we had applied to the head and entire spine. In both of Dr. Carpenter's cases, the pulse fell immediately after the application of the ice—in case 1st, from 110 to 75 ; and in case 2d, from 120 to 100—and every urgent symptom was relieved. In our case, the ice had no effect whatever, the pulse continuing at

120, without the slightest yielding of the spasm. We continued the application of the ice, (removing it for a few moments every ten minutes,) for more than an hour. During the night and next day chloroform was freely used, which mitigated his sufferings, without affecting their cause, and on the following day he died. We should have mentioned that actual cautery was freely applied to the lacerated toe.

Case 2d.—On the 1st day of February last, our friend, Dr. Byrne, sent for us twelve miles in the country to see a patient of his suffering with Traumatic Tetanus. The patient was a heavy, well-made, muscular negro man. He had been unfortunate in mashing the end of the index finger of the right hand nearly three weeks before. He had taken some cathartic medicine, which had not operated. His pulse was 110, and hard. It was impossible for him to lie down, and he was propped up in a sitting posture against a plank. The spasm was occasionally frightful, when he would become bathed in a cold perspiration, and the pulse become so rapid as to be counted with difficulty. Between the severe paroxysms the anterior and posterior muscles of the body were permanently rigid, swaying the body to and fro, as either set gained the ascendency ; and when balanced in their antagonism, keeping the body bolt upright against the plank, his occiput pressed firmly against it, with a slight conquassatory motion of the head, from alternating spasms of the sterno-cleido-mastoid muscles.

The lacerated finger presented no evidence of granulation ; and a ragged gash in its pulp, extending the whole length of the first phalanx and over-lapping a third of the second, looked dry and callous.

Upon consultation, we agreed to apply the actual cautery to the injured finger, to encourage the medicine, previously given, to operate on the bowels, and then to give opium in liberal doses, in combination with small doses of calomel, at intervals of four hours. Dr. Byrne suggested the wine of colchicum as very certain, after a mercurial, to produce purging, and forty drops were immediately given him, which dose was repeated in a few hours. Copious purging resulted, and with it so alarming an increase of spasm, as to threaten immediate dissolution. I noticed, moreover, that when the cautery was applied to the finger, the right leg was immediately seized with a violent spasm, while the left remained unaffected. After pretty thorough purging, four grains of opium and ten of calomel were given him, which dose was repeated at intervals of four hours. When bordering on narcotism, the opium was reduced in quantity to two or even one grain. Boiling spirit of turpentine was applied next day to the finger.

On the third day from the commencement of this treatment, ptyalism appeared, and the spasm gradually abated. The mercury was now withdrawn, but the opium in grain doses continued. This man entirely recovered.

From the fortunate result from the application of ice in Dr. Carpenter's cases, we were led to entertain a very favorable opinion of its power to control tetanic spasm ; and while we did not advise it in the second case, we hope its efficacy may be tested by farther trial. There is unquestionably a tetanic atmospheric constitution existing at this time, and injuries, which under other circumstances would be unworthy of notice, demand the careful, supervisory care of the practitioner.

While upon the subject of Tetanus, we beg permission of the reader to introduce another, though artificial case.

Mrs. M., of this city, recently married, had for a few consecutive evenings complained of pain in the right extremity of the forehead, involving the eye of that side, which, during the pain, would weep an abundance of hot tears. When the pain was off, she was well, and continued in the discharge of her domestic duties. On Sabbath day, 15th January last, her husband having leisure, borrowed a newspaper to find out who was the best docter in the city, and his eye fell upon the advertisement of one of the class who promised, "No cure, no pay;" and the "Special Notices" were eminently satisfactory. Thus assured, without consulting the patient, the husband found the office of the doctor, related the case of his wife, and returned with "two small yellowish white pills," which the doctor directed should be taken immediately. When the husband returned, the wife, with her mother, was engaged in preparing supper. He insisted upon her taking the pills immediately, remarking with evident satisfaction that they were to cost nothing if they failed to cure her head. The wife begged to be excused, alledging that she never felt better; and her mother interposed for her, but the husband was inexorable, and the pills were swallowed. In just one hour afterwards she fell down on the floor, not insensible, but rigid with spasm. My friend, Dr. J. S. Duval, was immediately summoned, and upon reaching the house, dispatched a messenger for me. Learning the history, and discovering the spasm was indisputably tetanic, I had not a doubt that she was poisoned with strychnine; and as this fact imparted a greater interest to the case, I, with Dr. Duval's concurrence, demanded aditional counsel, and at my suggestion, my friend, Dr. Porter was called in. He agreed with us that the case was one of artificial tetanus, and doubtless produced by strychnine. The spasm increased in severity, and the patient suffered inconceivable agony. The poor old mother would rush to the bedside of her only child, wring her withered hands with auguish, cry out at the top of her voice, "Poisoned! Poisoned!" and find temporary repose in insensibility and the floor. The patient had great thirst, but water no sooner touched her lip than general spasm seized her.

A napkin, folded in a narrow strip, and of sufficient length to reach from the head to the sacrum, was saturated with chloroform and applied to the whole length of the spine, and covered with a dry one, folded in like manner, to prevent evaporation. Our object was to produce rapid vesication; but although the chloroform was several times applied, it did not succeed in this object. Five hours of severe suffering having elapsed, we suggested, (Dr. P. having left,) a grain of Ipecac every half hour, if we could possibly get it in the stomach, until some decided effect should result. Our object was not to vomit, but to nauseate. Tonicity is not irritability, however intimate the relation between these primary elements of function. Tonicity is lowered by nausea—irritability is increased by emetics. Tetanic spasm is characterized by its tonicity; and it was this element which we desired to reach. We have said that her lip no sooner touched the water in a glass, than violent spasm ensued; yet, with some difficulty, we got a grain of ipecac in a teaspoonful of water in her mouth, which she swallowed. We had stirred up 12 grains with 12 teaspoonfuls of water, with the determination of repeating the doses as above, and patiently watching the effect. Nausea came with the third dose, and the tetanic phenomena were immediately mitigated. The intervals were lengthened to an hour between the doses, and finally to two hours. The spasm entirely left her in the course of four hours, when she slept pretty soundly for four hours, awaking free from tetanus, but with much muscular soreness, which subsided in a few days, leaving her well. In justice to the doctor, we must add, that she was cured of her neuralgia.—*Nashville Jour*

